# Reduced White Matter Integrity in Patients With End-Stage and Non-end-Stage Chronic Kidney Disease: A Tract-Based Spatial Statistics Study

**DOI:** 10.3389/fnhum.2021.774236

**Published:** 2021-12-10

**Authors:** Yuhan Jiang, Qiuyi Gao, Yangyingqiu Liu, Bingbing Gao, Yiwei Che, Liangjie Lin, Jian Jiang, Peipei Chang, Qingwei Song, Weiwei Wang, Nan Wang, Yanwei Miao

**Affiliations:** ^1^Department of Radiology, The First Affiliated Hospital of Dalian Medical University, Dalian, China; ^2^Department of Nephrology, The First Affiliated Hospital of Dalian Medical University, Dalian, China; ^3^Department of Radiology, The Third People’s Hospital of Dalian, Dalian, China; ^4^Philips Healthcare China, Beijing, China

**Keywords:** tract-based spatial statistics, chronic kidney disease, end-stage renal disease, uric acid, phosphate

## Abstract

**Background and Purpose:** Reduced white matter (WM) integrity has been implicated in chronic kidney disease (CKD), especially in end-stage renal disease (ESRD). However, whether the differences in WM abnormalities exist in ESRD and non-end-stage CKD (NES-CKD) remains unclear. Hence, this study aimed to investigate the WM microstructural changes between the two stages using diffusion tensor imaging (DTI) and explore the related influencing factors.

**Methods:** Diffusion tensor imaging’ images were prospectively acquired from 18 patients with ESRD, 22 patients with NES-CKD, and 19 healthy controls (HCs). Tract-based spatial statistics (TBSS) was performed to assess the voxel-wise differences in WM abnormalities among the three groups. The relationships between DTI parameters and biochemical data were also analyzed.

**Results:** Compared with NES-CKDs, FA value was significantly decreased, and AD value increased in ESRDs mainly in brain regions of bilateral anterior thalamic radiation (ATR), the genu and body of corpus callosum (CC), bilateral anterior corona radiata, superior corona radiata, and superior longitudinal fasciculus. Besides, extensive and symmetrical deep WM damages were observed in patients with ESRD, accompanied by increased MD and RD values. Multiple regression analysis revealed that uric acid and serum phosphorus level can be used as independent predictors of WM microstructural abnormalities in clusters with statistical differences in DTI parameters between ESRD and NES-CKD groups.

**Conclusion:** In the progression of CKD, patients with ESRD have more severe WM microstructural abnormalities than NES-CKDs, and this progressive deterioration may be related to uric acid and phosphate levels.

## Introduction

Chronic kidney disease (CKD) is defined as decreased kidney function shown by an estimated glomerular filtration rate (eGFR) of less than 60 ml/min per 1.73 m^2^ or the presence of albuminuria with at least 3-month duration (Zhang L. et al., 2012; Webster et al., 2017). End-stage renal disease (ESRD) occurs when CKD progresses to the eGFR of < 15 ml/min/1.73 m^2^. Continuous renal replacement therapy is essential to maintain the health of patients with ESRD, and hemodialysis accounts for the largest proportion in China (Navva et al., 2015). About 10–40% of patients with CKD had impaired cognitive function (Sarnak et al., 2013) and gradually progresses with the decline of kidney function. Patients at stage 4 have almost two times the memory and attention deficits compared with stage 3 (Egbi et al., 2015). In addition, patients with ESRD treated with hemodialysis show a 26–60% prevalence of mild cognitive impairment. The neurocognitive decline is most likely to be related to brain structure damage, especially white matter (WM) abnormalities (Vogels et al., 2012). Many studies have shown that WM abnormalities may appear earlier than brain atrophy and are more sensitive to early marker (Bartzokis, 2011; Metzler-Baddeley et al., 2019). In addition, more severe CKD may lead to more severe WM damage, which is associated with cognitive impairment (Liu et al., 2020). Therefore, WM integrity may be a more suitable biomarker for brain changes in patients with CKD.

Diffusion tensor imaging (DTI), a non-invasive neuroimaging technique, measures the diffusion tensor of water molecules and provides a detailed assessment of fiber tracts, which has been widely applied in microstructural WM integrity assessment (Hagmann et al., 2006; Nucifora et al., 2007). Therefore, patients with CKD can apply DTI technology for regular follow-up to track the changes in WM microstructure and conduct a detailed assessment of cognitive-related microstructure impairment. Some other more advanced dMRI techniques, such as diffusion kurtosis imaging (DKI) (Fieremans et al., 2011), neurite orientation dispersion and density imaging (NODDI) (Zhang H. et al., 2012), and fixel-based analysis (FBA) (Raffelt et al., 2015), provide additional and richer indicators to detailed evaluate the microstructure characteristics of tissues.

Previous studies based on DTI have found that patients with ESRD have abnormal WM integrity in multiple regions of the brain (Kong et al., 2014; Zhang et al., 2015; Yin et al., 2018; Chou et al., 2019). However, studies on the changes in WM integrity of patients with non-end-stage CKD (NES-CDK) are still limited. So far, only one study by Liu et al. (Liu et al., 2020) revealed that adult patients with CKD at stage 4 have reduced integrity of WM tracts in the corpus callosum (CC), anterior thalamic radiation (ATR), inferior fronto-occipital fasciculus (IFOF), and inferior longitudinal fasciculus (ILF). To rule out the effects of long-term hemodialysis, their study only included patients with CKD at stages 3–4. In fact, a report of longitudinal WM alterations in patients with ESRD showed that the toxic effect of ESRD itself may be the major factor of poor WM integrity (Chou et al., 2019).

Although WM microstructural abnormality in patients with CKD has been suggested, no studies have yet reported the direct comparison of DTI parameters (fractional anisotropy [FA], mean diffusivity [MD], axial diffusivity [AD], and radial [RD] diffusivity) between patients with ESRD and NES-CKD using tract-based spatial statistics (TBSS) analysis, to the best of our knowledge. TBSS is an advanced hypothesis-free method for analyzing whole-brain WM fiber tracts, which has been widely used in studies of various neurological diseases (Smith et al., 2006; Chen et al., 2018; Lim et al., 2019; Mei et al., 2020; Yamada et al., 2020). Compared with voxel-based analysis (VBA) using statistical parametric mapping, TBSS improves the registration sensitivity and accuracy based on the diffusion data projected onto a single, averaged FA skeleton located at the center of major cerebral WM pathways. TBSS also overcomes the personal evaluation bias in methods based on regions of interest (Smith et al., 2006; Qi et al., 2013).

This study applies TBSS to examine the microstructural abnormalities of the whole-brain WM skeleton among participants with ESRD, NES-CDK, and healthy controls (HCs) to accurately explore the characterization of WM alterations during CKD progression. The DTI metrics used in this study include FA, MD, AD, and RD, which may provide an overall characterization of WM alterations in patients with CKD. We hypothesize that WM microstructural alterations existed differences in the patients with ESRD and NES-CKD. We also explored the relationships among these DTI parameters with the clinical and laboratory data, and also cognitive function in participants with ESRD.

## Materials and Methods

### Participants

This study was approved by the Local Ethics Committee of the First Affiliated Hospital of Dalian Medical University. Informed consents were obtained from patients or legal guardians before the study. CKD diagnosis was confirmed by a nephrologist according to the kidney disease outcomes quality initiative (K/DOQI) classification, with < 15 and 15–59 ml/min/1.73 m^2^ classified as ESRD and NES-CDK, respectively. Other inclusion criteria for patients with ESRD and NES-CDK were as follows: (1) maintenance hemodialysis (3–4 times per week) for at least 3 months and no dialysis, respectively; (2) age > 18 years; and (3) right-handedness. Exclusion criteria included the following: (1) had a history of traumatic brain injury, psychiatric diseases, or other neurological disorders (e.g., infarction); (2) recipient of renal transplant; and (3) contraindications for MRI examination (e.g., claustrophobia, pacemaker).

Between April 2019 and May 2021, 45 patients diagnosed with CKD were prospectively recruited in this study. Patients with poor image quality (*n* = 3) and with claustrophobia (*n* = 2) were excluded. Therefore, 40 patients with CKD, including 18 patients with ESRD and 22 with NES-CKD at stages 1–4, were enrolled in the final analysis. Nineteen HCs (right-handedness) were recruited with similar age, sex, body mass index (BMI), and education level to the participants with CKD. The exclusion criteria for HCs were traumatic brain injury, mental disorder, and neurological disorders.

### Neurocognitive Assessments

Among 40 patients with CKD, only 12 ESRD participants completed cognitive assessment [Beijing revised version Montreal Cognitive Assessment (MoCA)] before MR data acquisition. MoCA is a fast, comprehensive, deliberate, and sensitive neurocognitive assessment tool, which has previously been applied in the CKD population (Tiffin-Richards et al., 2014; Liu et al., 2020).

### Clinical Data and Laboratory Tests

All patients with CKD underwent clinical data collection (course of disease, admission blood pressure) and several biochemical tests, including serum creatinine (Scr), creatinine (Cre), serum urea (Urea), uric acid (UA), cystatin C (Cys C), cholesterol (CHOL), homocysteine (HCY), low-density lipoprotein (LDH), high-density lipoprotein (HDL), triglyceride (TG), serum kalium (K), serum natrium (Na), serum calcium (Ca), serum phosphorus (P), and parathyroid hormone (PTH) levels before MR data acquisition. No biochemical test was performed on the participants in the HC group.

### Diffusion Tensor Imaging

#### Diffusion Tensor Imaging Data Acquisition

Diffusion-weighted images were obtained with a 3.0T MRI scanner (Ingenia CX, Philips Healthcare, Best, Netherlands) equipped with a 32-channel phased-array head coil, using a single-shot echo-planar imaging (SS-EPI) sequence. The parameters were as follows: 64 non-collinear spatial directions at *b* value = 1,000 s/mm^2^, one baseline image at *b* = 0 s/mm^2^, TR/TE = 6,000 ms / 92 ms, voxel size = 2 mm × 2 mm × 2 mm, matrix size = 128 × 128, field of view = 256 mm × 256 mm, slice thickness = 2 mm, without a slice gap. A total of 68 axial slices were collected, covering the whole brain, and the duration of the DTI scan was 6 min and 46 s.

#### Diffusion Tensor Imaging Data Preprocessing

Diffusion tensor imaging data were processed using the Functional MRI of the Brain (FMRIB) Software Library (FSL) version 5.0.9^1^ (Smith et al., 2004). Eddy current-induced distortions and motion artifacts were corrected by registering each diffusion-weighted image to the non-diffusion weighted volume (b0 image) using the affine alignment (Andersson and Sotiropoulos, 2016). All images were visually inspected before and after corrections. “Brain Extraction Tool (BET)” inside the FSL package was used to extract a brain mask from the eddy corrected image to remove the skull and non-brain tissue (Smith, 2002). Diffusion tensor at each voxel was fitted using the DTIFIT tool to generate FA, MD, and eigenvalue (λ1, λ2, λ3) maps. Axial (AD = λ1) and radial [RD = (λ2 + λ3)/2] diffusivity maps were then calculated from these eigenvalues.

#### Tract-Based Spatial Statistics Analysis

Voxel-wise statistical analysis of the FA maps was performed using the TBSS toolbox in FSL (Smith et al., 2004, 2006). All FA maps were spatially aligned to a 1 mm × 1 mm × 1 mm FMRIB58 FA standard space using a non-linear registration algorithm. The aligned FA maps were averaged to create a mean FA image and then skeletonized to generate a mean FA skeleton, which represents the center of all WM fiber tracts common to all participants. The FA threshold for the skeletonization was 0.20 to exclude gray matter and cerebrospinal fluid interference and also intersubject variability. Aligned FA maps for all subjects were then projected onto this skeleton. The same FA transformation was then also applied to MD, AD, and RD images for statistical analysis.

To estimate the voxel-wise FA, MD, AD, and RD differences among the three groups, individual skeleton images were inputted to the general linear model (GLM) analysis, adjusting for age, sex, years of education, and BMI as covariates. Non-parametric permutation-based testing was performed using randomize in FSL (Winkler et al., 2014). One-way analysis of covariance (ANOVA) was performed with one *F*-test for the overall group effect on diffusion parameters (FA, AD, RD, and MD) and six contrasts for the individual comparisons of voxel-wise diffusion parameters between the groups. Results are reported at the *p* < 0.05 level after 5,000 permutations using permutation-based non-parametric inference, with threshold-free cluster enhancement (TFCE) and family-wise error (FWE) rate correction for multiple comparisons (Smith and Nichols, 2009).

The FSL’s cluster was used to identify statistically significant (*p* < 0.05) clusters followed by an atlas query to describe the localization of all the anatomical clusters using the John Hopkins University (JHU)—International Consortium of Brain Mapping DTI-81 WM labels and JHU white matter tractography atlas template. DTI parameters in each significant cluster were then extracted from the skeletonized TBSS image of each participant.

### Statistical Analysis

Statistical analysis was carried out using IBM SPSS software, version 22.0. One-way ANOVA was used to compare the ages and BMI of the participants among the three groups. A Kruskal–Wallis test was used to compare the years of education. Data without normal distributions were analyzed using non-parametric tests. Categorical variable analyses were analyzed by chi-squared test. The clinical features between the ESRD and CKD groups were performed using the independent sample *t*-tests.

For normally distributed data, the partial correlation was used to assess the association between the extracted DTI parameters and clinical data, adjusting for the same covariates as above. Non-normally distributed data were analyzed using Spearman’s correlation coefficient. Statistical significance was defined as two-tailed *p* < 0.05. Bonferroni’s correction was applied for multiple testing.

## Results

### Demographic and Clinical Characteristics

The demographic and clinical characteristics of the subjects from the ESRD, NES-CKD, and HC groups are presented in Table 1. There was no significant difference in gender, age, BMI, and years of education (*p* > 0.05). The ESRD group showed a significantly increased Cys C, Urea, Cre, K, Na, P, and PTH compared with the NES-CKD group (*p* < 0.01).

**TABLE 1:** Demographic and clinical characteristics.

	ESRD (*n* = 18)	NES-CKD (*n* = 22)	HC (*n* = 19)	t/Z/F/χ2	*p*-value
Gender (M/F)	10 / 8	11 / 11	8 / 11	0.679	0.712
Age, years	57.28 ± 12.22	54.68 ± 13.62	56.05 ± 11.65	0.212	0.810
BMI, Kg/m^2^	23.63 ± 2.78	25.57 ± 4.27	24.25 ± 2.42	1.799	0.175
Education, years, M (IQR)	13 (9.75, 15)	12 (9, 15)	12 (12, 15)	0.275	0.871
MoCA, M (IQR)	26 (25, 28)	–	23.5 (22, 24.25)	−2.894	**0.004** *
Stage, *n* (%)	Stages 5, 18 (100)	Stages 1, 6 (27.27)	–		
		Stages 2, 4 (18.18)			
		Stages 3, 6 (27.27)			
		Stages 4, 6 (27.27)			
**Basic admissions**					
Hypertension, *n* (%)	13 (72.2)	13 (59.1)	–	6.599	0.298
Diabetes Mellitus, *n* (%)	13 (72.2)	13 (59.1)	**–**	0.750	0.298
History of smoking, *n* (%)	4 (22.2)	7 (31.8)	**–**	0.457	0.377
History of drinking, *n* (%)	3 (16.7)	2 (22.0)	**–**	0.519	0.402
SBP, mmHg, M (IQR)	145 (130, 153.75)	130 (120, 140)	**–**	−1.468	0.155
DBP, mmHg, M (IQR)	80 (80, 80)	80 (76.25, 90)	–	−0.302	0.798
**Biochemical parameters**					
Glu, mmol/L, M (IQR)	4.58 (4.42, 5.54)	5.22 (4.76, 5.66)	**–**	−1.115	0.274
Cys C, mg/L, M (IQR)	7.15 (6.73, 7.81)	1.81 (1.16, 2.40)	**–**	−5.356	**0.000** *
CHOL, mmol/L, M (IQR)	4.75 (4.38, 5.02)	5.2 (4.71, 6.08)	**–**	−1.713	0.089
TG, mmol/L, M (IQR)	1.48 (1.17, 1.91)	1.59 (1.14, 2.51)	**–**	−0.381	0.717
HDL, mmol/L	1.14 ± 0.28	1.05 ± 0.40	–	−0.856	0.397
LDL, mmol/L	2.62 ± 0.65	3.05 ± 0.81	**–**	1.785	0.082
Urea, mmol/L	24.67 ± 5.68	11.35 ± 6.22	**–**	−7.065	**0.000** *
UA, μmol/L	403.33 ± 104.41	412.91 ± 120.26	**–**	0.266	0.792
K, mmol/L	4.95 ± 0.66	4.10 ± 0.53	**–**	−4.523	**0.000** *
HCY, mmol/L, M (IQR)	23.41 (19.12, 27.47)	19.43 (13.25, 22.54)		−1.821	0.070
Cre, μmol/L, M (IQR)	1,008.5 (829.75, 1,121.5)	107.5 (79, 186.25)		−5.383	**0.000** *
Na, mmol/L, M (IQR)	138 (136.25, 139)	141 (139.25, 141)		−3.520	**0.000** *
Ca, mmol/L, M (IQR)	2.25 (2.21, 2.28)	2.22 (2.10, 2.26)		−1.415	0.163
P, mmol/L, M (IQR)	1.76 (1.29, 2.04)	1.28 (1.37)		−2.746	**0.005** *
PTH, mmol/L, M (IQR)	191.75 (75.27, 478.18)	54.63 (32.33, 108.1)		−2.528	**0.011** *

*ESRD, end-stage renal disease; NES-CKD, non-end-stage chronic kidney disease; HC, health controls; n, number; BMI, body mass index; MoCA, Montreal Cognitive Assessment; SBP, systolic blood pressure; DBP, diastolic blood pressure; Glu, glucose; Cys C, cystatin C; CHOL, cholesterol; TG, triglyceride; HDL, high-density lipoprotein; LDH, low-density lipoprotein; Urea, serum urea; UA, uric acid; K, kalium; HCY, homocysteine; Cre, serum creatinine; Na, serum natrium; Ca, serum calcium; P, serum phosphorus; PTH, parathyroid hormone.*

*M, median; IQR, interquartile range; Values are presented as the mean ± standard deviation (SD), n (%), or M (IQR).*

* and bold values indicates a statistical difference between groups, p < 0.05.

### Tract-Based Spatial Statistics Results Between Groups

#### ANCOVA

An ANCOVA revealed a statistically significant effect for FA, MD, AD, and RD across ESRD, NES-CKD, and HC groups (*p* < 0.05, TFCE-corrected, Supplementary Figure 1).

#### ESRD vs. NES-CKD

**FIGURE 1:**
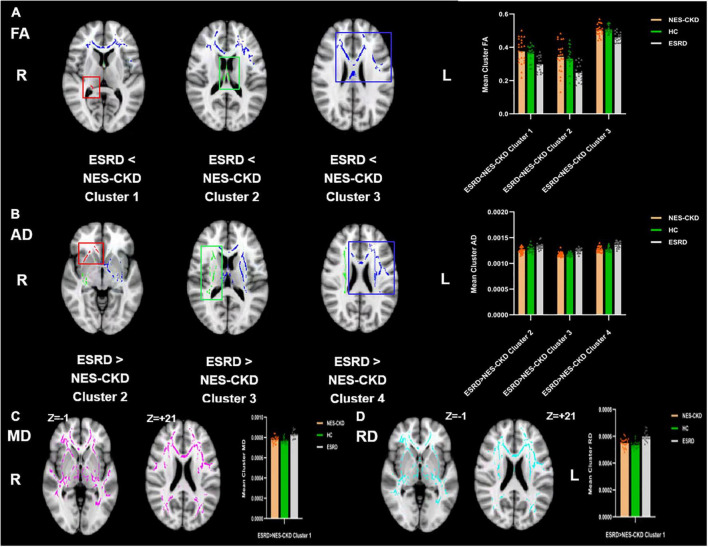
Voxel clusters in which patients with ESRD exhibited decreased FA or increased AD, MD, or RD than NES-CKD. **(A)** Three independent clusters (red for Cluster 1; green for Cluster 2; blue for Cluster 3) with lower FA values in ESRD vs. NES-CKD. **(B)** Three independent clusters (red for Cluster 2; green for Cluster 3; blue for Cluster 4) with higher AD values in ESRD vs. NES-CKD. **(C)** One independent cluster (pink) with higher MD values in ESRD vs. NES-CKD. **(D)** One independent cluster (light blue) with higher RD values in ESRD vs. NES-CKD. The bar plots represent the corresponding mean diffusion metrics for each group. The triangle symbols indicate the data points of each participant in each group. ESRD, end-stage renal disease; NES-CKD, non-end-stage chronic kidney disease; HC, healthy control; FA, fractional anisotropy; MD, mean diffusivity; AD, axial diffusivity; RD, radial diffusivity.

We identified three independent clusters with statistically significant differences in FA and AD values and one independent cluster with a significant difference in MD and RD values between ESRD and NES-CKD groups. When compared to patients with NES-CKD, these clusters showed decreased FA or increased MD, AD, or RD in the ESRD group. Additionally, in these clusters, the average diffusion index values in the NES-CKD and HC groups were roughly similar (Figure 1).

**FIGURE 2:**
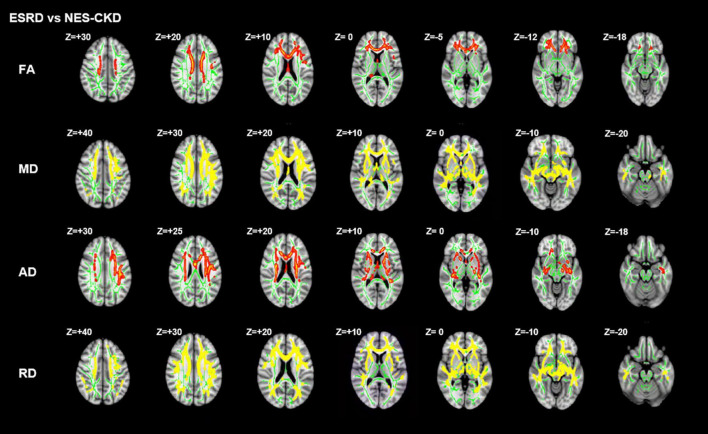
*Post hoc* analyses result between patients with ESRD and NES-CKD. Green represents the mean FA skeleton of all subjects. Red–yellow represent regions with significant statistical values (*p* < 0.05, TFCE-corrected). ESRD, end-stage renal disease; NES-CKD, non-end-stage chronic kidney disease; FA, fractional anisotropy; MD, mean diffusivity; AD, axial diffusivity; RD, radial diffusivity.

Figure 2 showed the more detailed locations of *post hoc* analyses between ESRD and NES-CKD groups. We found significantly reduced FA values in the ESRD group compared to the NES-CKD group, mainly in bilateral ATR, the genu, and body of CC, bilateral anterior corona radiata (ACR), bilateral superior corona radiata, bilateral IFOF, and left superior longitudinal fasciculus (SLF) (*p* < 0.05, TFCE-corrected, Table 2). Besides, significantly increased AD values in patients with ESRD were confined to genu and body of CC, right ACR, bilateral ATR, right internal capsule, bilateral external capsule, bilateral corticospinal tract, bilateral corona radiata, left SLF, left IFOF, and left IFOF (*p* < 0.05, TFCE-corrected, Table 3). In addition, we observed extensive and symmetrical deep WM damage in patients with ESRD, accompanied by increased MD and RD values, including CC, bilateral corona radiata, bilateral ATR, bilateral IFOF, bilateral SLF, bilateral internal capsule, and bilateral external capsule (Supplementary Tables 1, 2).

**TABLE 2:** Cluster sizes and locations for voxels with significantly reduced FA in ESRD vs. NES-CKD groups.

Cluster Number	JHU WM tractography atlas	JHU ICBM-DTI-81 WM labels	Voxel coordinates of Local maxima (MNI coordinates)			Voxels	Z-score	*p*-value
			X	Y	Z			
1	Anterior thalamic radiation R:4.38596	Fornix (cres) / Stria terminalis R:7.01754	18	−32	10	57	−1.69	0.046
2	Anterior thalamic radiation L:2.08997							
		Fornix (column and body of fornix):25.2595	1	−1	15	289	−1.80	0.036
	Anterior thalamic radiation R:3.62976							
3	Anterior thalamic radiation L:2.05252	Genu of CC:11.5581						
	Anterior thalamic radiation R:1.746	Body of CC:22.6704						
	Forceps minor:13.8569	Anterior corona radiata R:9.6662						
	Inferior fronto-occipital fasciculus L:2.35653	Anterior corona radiata L:11.7756	10	30	7	9,197	−3.10	0.001
	Inferior fronto-occipital fasciculus R:2.15581	Superior corona radiata R:3.84908						
	Superior longitudinal fasciculus L:1.18321	Superior corona radiata L:3.32717						
	Uncinate fasciculus L:1.42014	Superior longitudinal fasciculus L:1.90279						

*MNI, Montreal Neurological Institute; L, abbreviation for the left hemisphere, R, abbreviation for the right hemisphere; JHU-WM tractography atlas, John Hopkins University white matter tractography atlas; JHU-ICBM-DTI-81 WM labels, John Hopkins University International Consortium of Brain Mapping DTI-81 WM labels; ESRD, end-stage renal disease; NES-CKD, non-end-stage chronic kidney disease; FA, fractional anisotropy.*

*The value after each region indicates the percentage probability of the cluster belonging to the given atlas label.*

*Any cluster with low voxels (<50) or region with low (<1%) probability has been excluded.*

**TABLE 3:** Cluster sizes and locations for voxels with significantly increased AD in ESRD vs. NES-CKD groups.

Cluster Number	JHU WM tractography atlas	JHU ICBM-DTI-81 WM labels	Voxel coordinates of Local maxima (MNI coordinates)			Voxels	Z-score	*p*-value
			X	Y	Z			
2	Forceps minor:15.1661	Genu of CC:28.9902	9	26	−5	307	−1.75	0.040
	Inferior fronto-occipital fasciculus R:15.5505	Anterior corona radiata R:19.544						
	Uncinate fasciculus R:8.3127	External capsule R:31.5961						
3	Anterior thalamic radiation R:5.10322	Anterior limb of internal capsule R:10.9053	25	−18	13	2,916	−2.41	0.008
	Corticospinal tract R:3.94753	Posterior limb of internal capsule R:7.13306						
	Inferior fronto-occipital fasciculus R:6.52092	Retrolenticular part of internal capsule R:10.3567						
		Anterior corona radiata R:11.2483						
		Superior corona radiata R:20.0274						
		Posterior corona radiata R:4.59534						
		Posterior thalamic radiation (include optic radiation) R:1.09739						
		External capsule R:10.8711						
4	Anterior thalamic radiation L:3.55331	Genu of CC:8.5528	−27	−30	16	7,822	−3.10	0.001
	Corticospinal tract L:2.61826	Body of CC:4.75582						
	Forceps minor:4.45513	Fornix (column and body of fornix):1.38072						
	Inferior fronto-occipital fasciculus L:3.18499	Anterior limb of internal capsule L:4.57683						
	Inferior longitudinal fasciculus L:1.10892	Posterior limb of internal capsule L:6.0854						
	Superior longitudinal fasciculus L:6.35093	Retrolenticular part of internal capsule L:3.15776						
	Superior longitudinal fasciculus (temporal part) L:2.93659	Anterior corona radiata L:6.28995						
		Superior corona radiata L:11.4804						
		External capsule L:8.09256						
		Superior longitudinal fasciculus L:7.60675						

*MNI, Montreal Neurological Institute; L, abbreviation for the left hemisphere, R, abbreviation for the right hemisphere; JHU-WM tractography atlas, John Hopkins University white matter tractography atlas; JHU-ICBM-DTI-81 WM labels, John Hopkins University International Consortium of Brain Mapping DTI-81 WM labels; ESRD, end-stage renal disease; NES-CKD, non-end-stage chronic kidney disease; AD, axial diffusivity.*

*The value after each region indicates the percentage probability of the cluster belonging to the given atlas label.*

*Any cluster with low voxels (<50) or region with low (<1%) probability has been excluded.*

#### NES-CKD vs. HC

**FIGURE 3:**
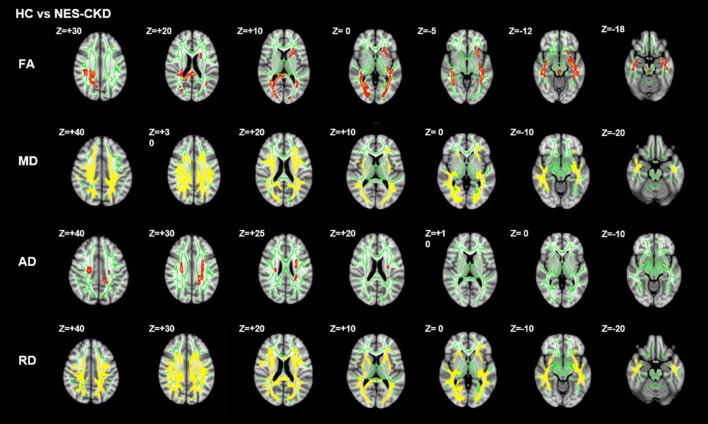
*Post hoc* analyses result between patients with NES-CKD and HCs. Green represents the mean FA skeleton of all subjects. Red–yellow represent regions with significant statistical values (*p* < 0.05, TFCE-corrected). NES-CKD, non-end-stage chronic kidney disease; HC, healthy control; FA, fractional anisotropy; MD, mean diffusivity; AD, axial diffusivity; RD, radial diffusivity.

We identified two independent clusters with statistically significant differences in FA values between the NES-CKD and HC groups. When compared to HC patients, all of these clusters showed decreased FA in the NES-CKD group (Figure 3).

**FIGURE 4:**
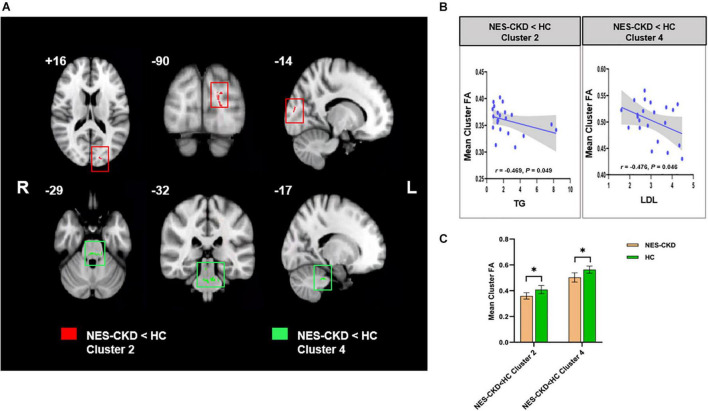
**(A)** Biochemical tests relevant clusters with statistically significant FA differences obtained from the NES-CKD and HCs. Cluster 2 is marked in red and Cluster 4 in green. **(B)** Relationship between mean cluster FA and TG (left) and LDL (right). **(C)** The bar plots represent the corresponding mean FA for each group. NES-CKD, non-end-stage chronic kidney disease; HC, healthy control; FA, fractional anisotropy. * indicates a statistical difference between the two groups, *p* < 0.05.

Figure 4 showed the detailed locations of *post hoc* analyses between NES-CKD and HC groups. We found lower FA and higher MD, AD, and RD values in patients with NES-CKD compared with HCs. FA decreased mainly in forceps major, middle cerebellar peduncle, right corticospinal tract, right SLF, left ATR, bilateral IFOF, left ACR, left anterior limb of the internal capsule, left posterior thalamic radiation, left external capsule, splenium of CC, and right posterior corona radiata. Refer to Supplementary Table 3 for detailed information. Increased AD values are mainly located in the bilateral corona radiata, the body of CC, and bilateral corticospinal tracts (Supplementary Table 4). In addition, MD and RD values showed extensive and symmetrical increases in the deep WM. Detailed results were listed in Supplementary Tables 5, 6.

### Correlation Analysis

In the NES-CKD group, partial correlation analysis was conducted between the clusters (with statistically significant differences between the NES-CKD and HC groups) and biochemical tests, and we only found mean FA values in two independent clusters (Cluster 2 and Cluster 4) negatively correlated with TG (*r* = −0.469, *p* = 0.049) and LDL (*r* = −0.476, *p* = 0.046), respectively (Figure 3). Obviously, they did not survive Bonferroni’s correction.

**FIGURE 5:**
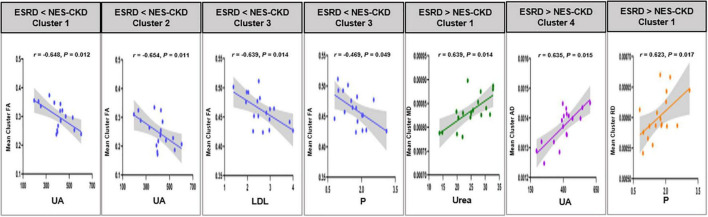
Relationship between mean cluster diffusion metrics and biochemical tests. UA, uric acid; LDH, low-density lipoprotein; P, serum phosphorus; Urea, serum urea. ESRD, end-stage renal disease; NES-CKD, non-end-stage chronic kidney disease; FA, fractional anisotropy; MD, mean diffusivity; AD, axial diffusivity; RD, radial diffusivity.

In the ESRD group, partial correlation analysis and multiple linear regression analysis revealed that UA level may serve as an independent predictor of mean FA changes in two clusters and mean AD changes in one cluster. Besides, LDL, P, and Urea levels may also be used as independent predictors of WM abnormalities in specific clusters with statistical differences in DTI parameters between ESRD and NES-CKD groups (Table 4). Figure 5 showed the above-mentioned results of the partial correlation analysis. Detailed correlations were displayed in Supplementary Figure 2. We did not find any relationship between mean diffusion metrics clusters and MoCA (*p* > 0.05).

**TABLE 4:** Multiple linear regression analysis.

	Clusters	β	*P*
FA	ESRD < NES-CKD Cluster 1		
	UA	−0.622	0.006
	ESRD < NES-CKD Cluster 2		
	UA	−0.568	0.014
	ESRD < NES-CKD Cluster 3		
	LDL	−0.529	0.007
	P	−0.480	0.013
MD	ESRD > NES-CKD Cluster 1		
	Urea	0.679	0.002
AD	ESRD > NES-CKD Cluster 4		
	UA	0.766	<0.001
RD	ESRD > NES-CKD Cluster 1		
	P	0.536	0.022

*UA, uric acid; LDH, low-density lipoprotein; P, serum phosphorus; Urea, serum urea. ESRD, end-stage renal disease; NES-CKD, non-end-stage chronic kidney disease; FA, fractional anisotropy; MD, mean diffusivity; AD, axial diffusivity; RD, radial diffusivity.*

## Discussion

In this study, we applied the TBSS method to investigate the difference in WM microstructure changes between patients with ESRD and NES-CKD and also NES-CKD and HCs. The relationship between DTI parameters and biochemical tests was also explored. The main findings of this work were as follows: (1) compared with HCs, FA values reduced, and MD, AD, and RD values increased significantly in patients with NES-CKD and ESRD, indicating the microstructural damage in patients with both ESRD and NES-CKD; (2) In comparison with patients with NES-CKD, patients with ESRD also showed a significant decrease in FA values and an increase in MD, AD, and RD values, which suggests that patients with ESRD have more serious WM microstructure abnormalities; (3) DTI parameters in some clusters were significantly correlated with biochemical tests, such as UA, Urea, and P, showing that these indicators may induce the fragility of WM microstructure in patients with ESRD.

At present, the research on the WM microstructure changes of NES-CKD by TBSS is very minimal, and only one study involving patients with stages 3 and 4 has been consulted (Liu et al., 2020). Similar to the results by Liu (Liu et al., 2020), our study also found decreased FA and increased MD values in multiple regions of patients with NES-CKD, especially in bilateral SLF, bilateral ILF, bilateral IFOF, CC, and bilateral corona radiata, in comparison with those of HCs.

Compared with the previous analyses based only on the FA and MD values, our study incorporated four diffusion metrics (FA, MD, AD, and RD), and thus the results can be more comprehensive. AD and RD indicate the degree of diffusion parallel to and perpendicular to the fiber orientation, respectively, and are sensitive to axon integrity and myelin damage. Our research showed that compared to healthy subjects, patients with NES-CKD have a broad and symmetrical increase in the RD value of WM microstructures, while the WM regions with increased AD values were relatively small. We speculate that in the progression of CKD, the reduction of FA is dominated by demyelination, and axonal injury only plays a small role. In addition, this study found that in regions where the MD values of patients with NES-CKD increased, the RD values also increased significantly. This finding has only been previously reported in ESRD (Chou et al., 2013). It is speculated that WM demyelination is the main neuropathy in patients with ESRD on long-term hemodialysis. But at present, changes in demyelination can also be observed in the progression of CKD. This also confirmed our above speculation.

In addition, in this study, reduced FA was observed in the middle cerebellar peduncle, which has not been mentioned previously. WM abnormalities in the infratentorial regions only have been reported in the study of ESRD after hemodialysis and were believed to be the results of transient edema and demyelination after hemodialysis (Tarhan et al., 2004; Lakadamyali and Ergün, 2011; Chou et al., 2013). However, the results of our study showed that there may also be changes in the microstructure of the subtentorial WM in patients with progressing CKD without dialysis, but the reason is still unclear and needs to be confirmed by larger samples or animal experiments.

Notably, in this study, we have found differences in WM characteristics between patients with ESRD and NES-CKD. To our knowledge, this is the first study applying TBSS to analyze the difference in WM microstructure between NES-CKDs and ESRDs.

In the ESRD group, although three independent clusters were identified with reduced FA values, and more than 96% voxels were located in Cluster 3, indicating that from non-end-stages to the end-stage, WM in patients with CKD has occurred large-scale alterations. Cluster 3 included voxels in the bilateral ATR, the genu and body of CC, bilateral ACR, bilateral superior corona radiata, bilateral IFOF, and left SLF. We found that most regions of this cluster were not shown in the different clusters between NES-CKD and HC groups, especially for genu and its extension radiating fibers (forceps minor) and body of CC and bilateral superior corona radiata, suggesting that the changes in WM characteristics in these regions only appear in the ESRD. As the largest WM tract, CC connects the bilateral hemispheres to realize their communication. *Post hoc* analysis showed decreased FA in the splenium of CC in patients with NES-CKD, while WM abnormalities in the genu and body of CC appeared in patients with ESRD, indicating that with the progress of CKD, the abnormality in WM microstructure of the CC is gradually developing. We also identified that WM damages appear mainly in superior brain portions of the ESRD group. Corona radiata includes the ascending and descending fibers of the thalamus and cerebral cortex and participates in various functions such as emotion, execution, and cognition (Drevets et al., 2008; Cui et al., 2020). Studies have confirmed that the incidence of depressive symptoms in patients with CKD is significantly higher than that of ordinary people (Fischer et al., 2012; Al-Ali et al., 2021). Therefore, we suspect that the WM damage of CR may be related to a certain degree of depressive symptoms in the enrolled patients. However, it is a pity that the depression has not been evaluated due to patients’ compliance, and follow-up studies are still needed.

Multiple regression analysis was conducted within the significant clusters and biochemical tests in the whole patients with ESRD to determine the impacts of biochemical indicators on WM microstructure. Excitingly, we identified the relationships between UA and mean FA or AD in three clusters demonstrating differences between ESRDs and NES-CKDs. Additionally, we also observed that these three clusters mainly include the region of ATR. ATR connects the mediodorsal and anterior thalamic nucleus with the frontal cortex and the anterior cingulate cortex and can also process information from the hippocampus (Setiadi et al., 2021), thus affecting cognitive function. Although there were literature supporting that UA can be beneficial to the pathological process of neurodegenerative diseases by reducing oxidative stress and free radicals (Kim et al., 2006; Lee et al., 2021), more and more studies have investigated the relationship between UA and cognitive impairment and found evidence about UA in exacerbating the deterioration of cognitive function (Beydoun et al., 2016; Li et al., 2021). Therefore, we speculate that UA may affect cognitive function through its effect on ATR. But this may be limited by the small sample size and the lack of correlation between UA and cognitive scores. Compared with the NES-CKD group, the phosphate and PTH levels in patients with ESRD were significantly higher, and the correlations observed in Table 4 indicated that the serum phosphate level may serve as an independent influencing factor of diffusion parameters in some significant clusters. This suggests an association between phosphate and WM abnormalities; however, little is known about the mechanism. When renal function is impaired, an increase in phosphate will cause the secretion of PTH but reduce the active form of vitamin D, which will induce secondary hyperparathyroidism (Tsuchiya and Akihisa, 2021). However, high PTH can affect the neurotransmission of the central nervous system and induce neurotoxicity (Wilmskoetter et al., 2019).

Several limitations of this study should be noted. First, the small sample size in this study may limit the generality of the results. Second, the enrolled patients with ESRD were all undergoing regular hemodialysis, and the effect of hemodialysis on WM abnormalities cannot be ruled out. Third, due to patient compliance issues, most patients have not completed the cognitive tests, which also has a certain impact on our results regarding cognitive function in patients. Fourth, patients with CKD at stages 1–4 were combined into one group for analysis. In the future, dynamic research on CKD can be considered to further reveal the changes in WM at different stages.

## Conclusion

In conclusion, the results of this study indicate that compared with patients in the progression of CKD, patients with ESRD have more severe WM microstructural abnormalities, and this progressive deterioration is related to uric acid and phosphate levels.

## Data Availability Statement

The original contributions presented in the study are included in the article/Supplementary Material, further inquiries can be directed to the corresponding author/s.

## Ethics Statement

The studies involving human participants were reviewed and approved by the Local Ethics Committee of the First Affiliated Hospital of Dalian Medical University. The patients/participants provided their written informed consent to participate in this study. Written informed consent was obtained from the individual(s) for the publication of any potentially identifiable images or data included in this article.

## Author Contributions

YJ and QG were the guarantor of integrity of the entire study. YJ, QG, YL, BG, YC, JJ, and PC performed the literature research. YJ, QG, YL, BG, YC, JJ, PC, QS, LL, NW, WW, and YM performed the clinical studies. YJ, QG, NW, and YM performed experimental studies and manuscript editing. YJ, QG, and JJ performed statistical analysis. All authors contributed to study concepts, study design, data acquisition, data analysis/interpretation, involved in manuscript drafting and manuscript revision for important intellectual content, approved the final version of the submitted manuscript, and agreed to ensure that any questions related to the work are appropriately resolved.

## Conflict of Interest

LL was employed by the company Philips Healthcare China. The remaining authors declare that the research was conducted in the absence of any commercial or financial relationships that could be construed as a potential conflict of interest.

## Publisher’s Note

All claims expressed in this article are solely those of the authors and do not necessarily represent those of their affiliated organizations, or those of the publisher, the editors and the reviewers. Any product that may be evaluated in this article, or claim that may be made by its manufacturer, is not guaranteed or endorsed by the publisher.

## Footnotes

1
http://fsl.fmrib.ox.ac.uk/fsl/

## Abbreviations

AD: axial diffusivity
ATR: anterior thalamic radiation
CKD: chronic kidney disease
CST: corticospinal
ESRD: end-stage renal disease
FA: fractional anisotropy
FDR: false discovery rate
LDL: low-density lipoprotein
P: serum phosphorus
MD: mean diffusivity
MoCA: Montreal Cognitive Assessment
RD: radial diffusivity
TBSS: tract-based spatial statistics
NES-CKD: non-end-stage chronic kidney disease
Urea: serum urea
UA: uric acid.
