# Supplementary material for: Reduced White Matter Integrity in Patients With End-Stage and Non-end-Stage Chronic Kidney Disease: A Tract-Based Spatial Statistics Study
Source: Front Hum Neurosci. 2021 Dec 10;15:774236. doi: 10.3389/fnhum.2021.774236 (PMC8709581; doi:10.3389/fnhum.2021.774236)
Supplement: Supplementary file 1 [file Data_Sheet_1.DOCX]

**Table S1. Cluster sizes and locations for voxels with significantly increased MD in ESRD vs NES-CKD group. The value after each region indicates the percentage probability of the cluster belonging to the given atlas label. Any cluster with low voxels (< 50) or region with low (<1%) probability has been excluded.**

| **Cluster Number** | **JHU-WM Tractography Atlas** | **JHU ICBM-DTI-81 White-Matter Labels** | **Voxel coordinates of Local maxima**  **(MNI coordinates)** | | | **Voxels** | **p-value** |
| --- | --- | --- | --- | --- | --- | --- | --- |
|  |  |  | **X** | **Y** | **Z** |  |  |
| 1 | Anterior thalamic radiation L:1.72496 | Genu of corpus callosum:4.1491 | -8 | 27 | 12 | 28705 | 0.003 |
|  | Anterior thalamic radiation R:1.6772 | Body of corpus callosum:6.89427 |  |  |  |  |  |
|  | Corticospinal tract L:1.05957 | Anterior limb of internal capsule R:1.12524 |  |  |  |  |  |
|  | Forceps minor:4.60442 | Anterior limb of internal capsule L:1.14266 |  |  |  |  |  |
|  | Inferior fronto-occipital fasciculus L:1.91817 | Posterior limb of internal capsule L:1.24717 |  |  |  |  |  |
|  | Inferior fronto-occipital fasciculus R:2.43463 | Retrolenticular part of internal capsule R:1.14963 |  |  |  |  |  |
|  | Inferior longitudinal fasciculus L:1.20101 | Anterior corona radiata R:4.07943 |  |  |  |  |  |
|  | Superior longitudinal fasciculus L:3.04229 | Anterior corona radiata L:4.07943 |  |  |  |  |  |
|  | Superior longitudinal fasciculus (temporal part) L:1.36774 | Superior corona radiata R:3.20502 |  |  |  |  |  |
|  |  | Superior corona radiata L:3.86344 |  |  |  |  |  |
|  |  | Sagittal stratum (include inferior longitidinal fasciculus and inferior fronto-occipital fasciculus) R:1.08343 |  |  |  |  |  |
|  |  | External capsule R:2.89497 |  |  |  |  |  |
|  |  | External capsule L:2.55356 |  |  |  |  |  |
|  |  | Superior longitudinal fasciculus L:2.98206 |  |  |  |  |  |

MNI, Montreal Neurological Institute; L, abbreviation for the left hemisphere, R, abbreviation for the right hemisphere; JHU-WM Tractography Atlas, John Hopkins University white matter tractography atlas; JHU-ICBM-DTI-81 White-Matter Labels, John Hopkins University International Consortium of Brain Mapping DTI-81 WM labels; ESRD, end stage renal disease; NES-CKD, non-end-stage chronic kidney disease; MD, mean diffusivity.

**Table S2. Cluster sizes and locations for voxels with significantly increased RD in ESRD vs NES-CKD group. The value after each region indicates the percentage probability of the cluster belonging to the given atlas label. Any cluster with low voxels (< 50) or region with low (<1%) probability has been excluded.**

| **Cluster Number** | **JHU-WM Tractography Atlas** | **JHU ICBM-DTI-81 White-Matter Labels** | **Voxel coordinates of Local maxima**  **(MNI coordinates)** | | | **Voxels** | **p-value** |
| --- | --- | --- | --- | --- | --- | --- | --- |
|  |  |  | **X** | **Y** | **Z** |  |  |
| 1 | Anterior thalamic radiation L:1.63418 | Genu of corpus callosum:4.10988 | 16 | 26 | 19 | 28249 | 0.003 |
|  | Anterior thalamic radiation R:1.54837 | Body of corpus callosum:8.064 |  |  |  |  |  |
|  | Forceps minor:5.46816 | Anterior corona radiata R:4.04616 |  |  |  |  |  |
|  | Inferior fronto-occipital fasciculus L:2.11983 | Anterior corona radiata L:4.52405 |  |  |  |  |  |
|  | Inferior fronto-occipital fasciculus R:2.31721 | Superior corona radiata R:1.85139 |  |  |  |  |  |
|  | Inferior longitudinal fasciculus L:1.25643 | Superior corona radiata L:2.37531 |  |  |  |  |  |
|  | Superior longitudinal fasciculus L:2.47867 | Posterior thalamic radiation (include optic radiation) L:1.26022 |  |  |  |  |  |
|  | Superior longitudinal fasciculus R:1.54313 | External capsule R:2.88506 |  |  |  |  |  |
|  | Superior longitudinal fasciculus (temporal part) L:1.13013 | External capsule L:1.97529 |  |  |  |  |  |
|  |  | Superior longitudinal fasciculus R:1.5682 |  |  |  |  |  |
|  |  | Superior longitudinal fasciculus L:2.04255 |  |  |  |  |  |

MNI, Montreal Neurological Institute; L, abbreviation for the left hemisphere, R, abbreviation for the right hemisphere; JHU-WM Tractography Atlas, John Hopkins University white matter tractography atlas; JHU-ICBM-DTI-81 White-Matter Labels, John Hopkins University International Consortium of Brain Mapping DTI-81 WM labels; ESRD, end stage renal disease; NES-CKD, non-end-stage chronic kidney disease; RD, radial diffusivity.

**Table S3. Cluster sizes and locations for voxels with significantly reduced FA in NES-CKD vs HC groups. The value after each region indicates the percentage probability of the cluster belonging to the given atlas label. Any cluster with low voxels (< 50) or region with low (<1%) probability has been excluded.**

| **Cluster Number** | **JHU-WM Tractography Atlas** | **JHU-ICBM-DTI-81 White-Matter Labels** | **Voxel coordinates of Local maxima**  **(MNI coordinates)** | | | **Voxels** | **p-value** |
| --- | --- | --- | --- | --- | --- | --- | --- |
|  |  |  | **X** | **Y** | **Z** |  |  |
| 2 | Forceps major:15.6977 |  | -13 | -91 | 10 | 86 | 0.047 |
|  | Inferior fronto-occipital fasciculus L:3.39535 |  |  |  |  |  |  |
|  | Inferior longitudinal fasciculus L:3.95349 |  |  |  |  |  |  |
| 3 | Corticospinal tract R:29 | Middle cerebellar peduncle:34.4828 | 8 | -18 | -31 | 145 | 0.039 |
|  |  | Corticospinal tract R:57.2414 |  |  |  |  |  |
|  |  | Cerebral peduncle R:6.89655 |  |  |  |  |  |
| 4 | Anterior thalamic radiation L:1.38693 | Middle cerebellar peduncle:19.0955 | 2 | -29 | -26 | 398 | 0.035 |
|  | Anterior thalamic radiation R:2.01759 | Pontine crossing tract (a part of MCP):40.9548 |  |  |  |  |  |
|  | Corticospinal tract L:2.25377 | Corticospinal tract R:1.25628 |  |  |  |  |  |
| 5 | Superior longitudinal fasciculus R:42.9248 | Superior longitudinal fasciculus R:63.7624 | 33 | -34 | 35 | 505 | 0.032 |
|  | Superior longitudinal fasciculus (temporal part) R:13.901 |  |  |  |  |  |  |
| 6 | Anterior thalamic radiation L:5.83207 | Anterior limb of internal capsule L:7.3255 | -33 | -7 | -12 | 2894 | 0.014 |
|  | Forceps major:1.53248 | Retrolenticular part of internal capsule L:3.6282 |  |  |  |  |  |
|  | Inferior fronto-occipital fasciculus L:21.2443 | Anterior corona radiata L:6.56531 |  |  |  |  |  |
|  | Inferior longitudinal fasciculus L:15.5425 | Posterior thalamic radiation (include optic radiation) L:16.586 |  |  |  |  |  |
|  | Superior longitudinal fasciculus L:2.06116 | Sagittal stratum (include inferior longitidinal fasciculus and inferior fronto-occipital fasciculus) L:14.8583 |  |  |  |  |  |
|  | Uncinate fasciculus L:5.33552 | External capsule L:16.1023 |  |  |  |  |  |
|  | Superior longitudinal fasciculus (temporal part) L:1.81064 | Superior fronto-occipital fasciculus (could be a part of anterior internal capsule) L:1.24395 |  |  |  |  |  |
| 7 | Forceps major:5.4709 | Splenium of corpus callosum:17.2336 | 32 | -63 | 0 | 3969 | 0.006 |
|  | Inferior fronto-occipital fasciculus R:10.9244 | Retrolenticular part of internal capsule R:1.25976 |  |  |  |  |  |
|  | Inferior longitudinal fasciculus R:11.4444 | Posterior corona radiata R:10.3553 |  |  |  |  |  |
|  |  | Posterior thalamic radiation (include optic radiation) R:14.7392 |  |  |  |  |  |
|  |  | Sagittal stratum (include inferior longitidinal fasciculus and inferior fronto-occipital fasciculus) R:9.97732 |  |  |  |  |  |

MNI, Montreal Neurological Institute; L, abbreviation for the left hemisphere, R, abbreviation for the right hemisphere; JHU-WM Tractography Atlas, John Hopkins University white matter tractography atlas; JHU-ICBM-DTI-81 White-Matter Labels, John Hopkins University International Consortium of Brain Mapping DTI-81 WM labels; HC, healthy control; NES-CKD, non-end-stage chronic kidney disease; FA, fractional anisotropy.

**Table S4. Cluster sizes and locations for voxels with significantly increased AD in NES-CKD vs HC groups. The value after each region indicates the percentage probability of the cluster belonging to the given atlas label. Any cluster with low voxels (< 50) or region with low (<1%) probability has been excluded.**

| **Cluster Number** | **JHU-WM Tractography Atlas** | **JHU ICBM-DTI-81 White-Matter Labels** | **Voxel coordinates of Local maxima**  **(MNI coordinates)** | | | **Voxels** | **p-value** |
| --- | --- | --- | --- | --- | --- | --- | --- |
|  |  |  | **X** | **Y** | **Z** |  |  |
| 4 | Corticospinal tract L:2.58125 | Superior corona radiata L:100 | -27 | -6 | 25 | 160 | 0.026 |
| 5 | Corticospinal tract R:18.9811 | Body of corpus callosum:7.57098 | 24 | -17 | 35 | 317 | 0.015 |
|  |  | Superior corona radiata R:87.0662 |  |  |  |  |  |
| 6 | Corticospinal tract L:6.80368 | Body of corpus callosum:2.24949 | -19 | -35 | 34 | 489 | 0.023 |
|  | Cingulum (cingulate gyrus) L:2.85481 | Splenium of corpus callosum:5.93047 |  |  |  |  |  |
|  |  | Superior corona radiata L:5.93047 |  |  |  |  |  |
|  |  | Posterior corona radiata L:39.2638 |  |  |  |  |  |
|  |  | Cingulum (cingulate gyrus) L:3.27198 |  |  |  |  |  |

MNI, Montreal Neurological Institute; L, abbreviation for the left hemisphere, R, abbreviation for the right hemisphere; JHU-WM Tractography Atlas, John Hopkins University white matter tractography atlas; JHU-ICBM-DTI-81 White-Matter Labels, John Hopkins University International Consortium of Brain Mapping DTI-81 WM labels; HC, healthy control; NES-CKD, non-end-stage chronic kidney disease; AD, axial diffusivity.

**Table S5. Cluster sizes and locations for voxels with significantly increased MD in NES-CKD vs HC groups. The value after each region indicates the percentage probability of the cluster belonging to the given atlas label. Any cluster with low voxels (< 50) or region with low (<1%) probability has been excluded.**

| **Cluster Number** | **JHU-WM Tractography Atlas** | **JHU ICBM-DTI-81 White-Matter Labels** | **Voxel coordinates of Local maxima**  **(MNI coordinates)** | | | **Voxels** | ***p*-value** |
| --- | --- | --- | --- | --- | --- | --- | --- |
|  |  |  | **X** | **Y** | **Z** |  |  |
| 1 | Anterior thalamic radiation L:1.4828 | Body of corpus callosum:2.66713 | -18 | -42 | 36 | 11398 | 0.007 |
|  | Corticospinal tract L:2.01491 | Splenium of corpus callosum:2.15827 |  |  |  |  |  |
|  | Cingulum (cingulate gyrus) L:1.23899 | Retrolenticular part of internal capsule L:1.84243 |  |  |  |  |  |
|  | Forceps major:1.47517 | Anterior corona radiata L:3.78137 |  |  |  |  |  |
|  | Inferior fronto-occipital fasciculus L:4.9034 | Superior corona radiata L:9.18582 |  |  |  |  |  |
|  | Inferior longitudinal fasciculus L:3.88384 | Posterior corona radiata L:4.32532 |  |  |  |  |  |
|  | Superior longitudinal fasciculus L:5.18556 | Posterior thalamic radiation (include optic radiation) L:4.14108 |  |  |  |  |  |
|  | Uncinate fasciculus L:1.25601 | Sagittal stratum (include inferior longitidinal fasciculus and inferior fronto-occipital fasciculus) L:2.09686 |  |  |  |  |  |
|  | Superior longitudinal fasciculus (temporal part) L:2.34295 | External capsule L:4.86928 |  |  |  |  |  |
|  |  | Cingulum (cingulate gyrus) L:1.83365 |  |  |  |  |  |
|  |  | Superior longitudinal fasciculus L:5.43078 |  |  |  |  |  |
| 2 | Corticospinal tract R:2.10479 | Body of corpus callosum:2.01934 | 19 | -88 | -1 | 12826 | 0.012 |
|  | Forceps major:1.45837 | Splenium of corpus callosum:1.8712 |  |  |  |  |  |
|  | Inferior fronto-occipital fasciculus R:4.08007 | Retrolenticular part of internal capsule R:1.55154 |  |  |  |  |  |
|  | Inferior longitudinal fasciculus R:4.37416 | Superior corona radiata R:9.42617 |  |  |  |  |  |
|  | Superior longitudinal fasciculus R:5.77374 | Posterior corona radiata R:2.799 |  |  |  |  |  |
|  | Superior longitudinal fasciculus (temporal part) R:2.29331 | Posterior thalamic radiation (include optic radiation) R:3.32917 |  |  |  |  |  |
|  |  | Sagittal stratum (include inferior longitidinal fasciculus and inferior fronto-occipital fasciculus) R:3.48511 |  |  |  |  |  |
|  |  | External capsule R:1.91798 |  |  |  |  |  |
|  |  | Cingulum (cingulate gyrus) R:1.32543 |  |  |  |  |  |
|  |  | Superior longitudinal fasciculus R:7.55497 |  |  |  |  |  |

MNI, Montreal Neurological Institute; L, abbreviation for the left hemisphere, R, abbreviation for the right hemisphere; JHU-WM Tractography Atlas, John Hopkins University white matter tractography atlas; JHU-ICBM-DTI-81 White-Matter Labels, John Hopkins University International Consortium of Brain Mapping DTI-81 WM labels; HC, healthy control; NES-CKD, non-end-stage chronic kidney disease; MD, mean diffusivity.

**Table S6. Cluster sizes and locations for voxels with significantly increased RD in NES-CKD vs HC groups. The value after each region indicates the percentage probability of the cluster belonging to the given atlas label. Any cluster with low voxels (< 50) or region with low (<1%) probability has been excluded.**

| **Cluster Number** | **JHU-WM Tractography Atlas** | **JHU ICBM-DTI-81 White-Matter Labels** | **Voxel coordinates of Local maxima**  **(MNI coordinates)** | | | **Voxels** | **p-value** |
| --- | --- | --- | --- | --- | --- | --- | --- |
|  |  |  | **X** | **Y** | **Z** |  |  |
| 1 | Anterior thalamic radiation L:1.09326 | Body of corpus callosum:3.31241 | 23 | -87 | 1 | 31065 | 0.004 |
|  | Forceps major:1.57467 | Splenium of corpus callosum:3.59569 |  |  |  |  |  |
|  | Inferior fronto-occipital fasciculus L:2.92229 | Retrolenticular part of internal capsule R:1.20071 |  |  |  |  |  |
|  | Inferior fronto-occipital fasciculus R:2.48984 | Anterior corona radiata L:1.81233 |  |  |  |  |  |
|  | Inferior longitudinal fasciculus L:2.21301 | Superior corona radiata R:2.95509 |  |  |  |  |  |
|  | Inferior longitudinal fasciculus R:2.12419 | Superior corona radiata L:2.62031 |  |  |  |  |  |
|  | Superior longitudinal fasciculus L:2.69261 | Posterior corona radiata R:1.51296 |  |  |  |  |  |
|  | Superior longitudinal fasciculus R:2.65028 | Posterior corona radiata L:1.31016 |  |  |  |  |  |
|  | Superior longitudinal fasciculus (temporal part) L:1.34006 | Posterior thalamic radiation (include optic radiation) R:1.99582 |  |  |  |  |  |
|  | Superior longitudinal fasciculus (temporal part) R:1.08878 | Posterior thalamic radiation (include optic radiation) L:2.10526 |  |  |  |  |  |
|  |  | Sagittal stratum (include inferior longitidinal fasciculus and inferior fronto-occipital fasciculus) R:1.72863 |  |  |  |  |  |
|  |  | Sagittal stratum (include inferior longitidinal fasciculus and inferior fronto-occipital fasciculus) L:1.44536 |  |  |  |  |  |
|  |  | External capsule R:1.50974 |  |  |  |  |  |
|  |  | External capsule L:2.54305 |  |  |  |  |  |
|  |  | Superior longitudinal fasciculus R:2.97441 |  |  |  |  |  |
|  |  | Superior longitudinal fasciculus L:2.84243 |  |  |  |  |  |

MNI, Montreal Neurological Institute; L, abbreviation for the left hemisphere, R, abbreviation for the right hemisphere; JHU-WM Tractography Atlas, John Hopkins University white matter tractography atlas; JHU-ICBM-DTI-81 White-Matter Labels, John Hopkins University International Consortium of Brain Mapping DTI-81 WM labels; HC, healthy control; NES-CKD, non-end-stage chronic kidney disease; RD, radial diffusivity.


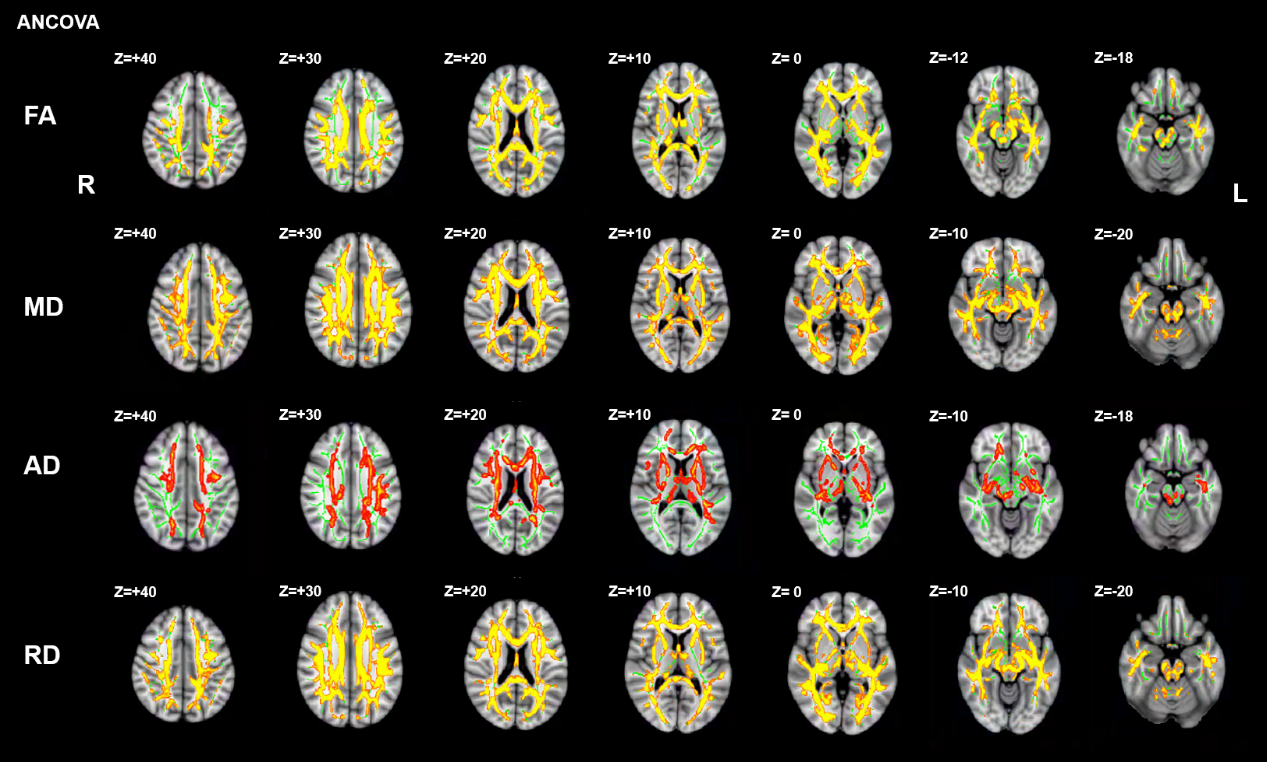


**Figure S1** FA, MD, AD and RD results of ANCOVA across ESRD, NES-CKD and HC groups. Green represents mean FA skeleton of all subjects. Red-yellow represent regions with significantly statistical values (*p* < 0.05, TFCE-corrected). ANCOVA, analysis of covariance; ESRD, end-stage renal disease; NES-CKD, non-end-stage chronic kidney disease; HC, healthy control; FA, fractional anisotropy; MD, mean diffusivity; AD, axial diffusivity; RD, radial diffusivity.


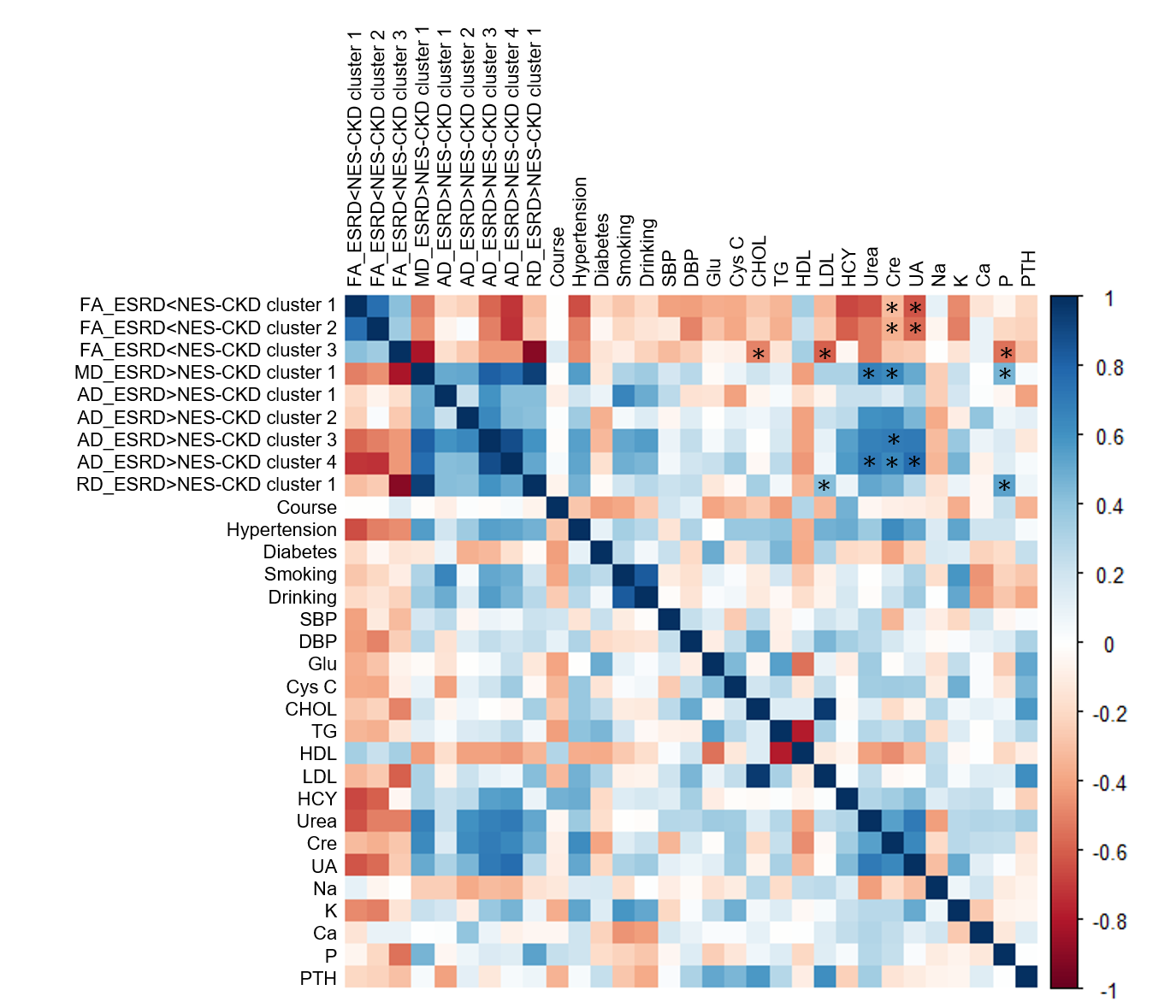


**Figure S2** Heatmap of correlations among mean cluster diffusion metrics and clinical and biochemical tests. * indicates *P* < 0.05. ESRD, end stage renal disease; NES-CKD, non-end stage chronic kidney disease; SBP, systolic blood pressure; DBP, diastolic blood pressure; Glu, glucose; Cys C, Cystatin C; CHOL, cholesterol; TG, triglyceride; HDL, high density lipoprotein; LDH, low density lipoprotein; Urea, serum urea; UA, uric acid; K, kalium; HCY, homocysteine; Cre, serum creatinine; Na, serum natrium; Ca, serum calcium; P, serum phosphorus; PTH, parathyroid hormone.
